# A New Customized Bioactive Glass Filler to Functionalize Resin Composites: Acid-Neutralizing Capability, Degree of Conversion, and Apatite Precipitation

**DOI:** 10.3390/jcm9041173

**Published:** 2020-04-19

**Authors:** Matej Par, Thomas Attin, Zrinka Tarle, Tobias T. Tauböck

**Affiliations:** 1 Department of Conservative and Preventive Dentistry, Center for Dental Medicine, University of Zurich, 8032 Zurich, Switzerland; 2 Department of Endodontics and Restorative Dentistry, School of Dental Medicine, University of Zagreb, 10000 Zagreb, Croatia

**Keywords:** dental resin composites, fluoride-containing bioactive glass, acid neutralization, degree of conversion, hydroxyapatite precipitation

## Abstract

This study introduced an experimental bioactive glass (BG) with a lower Na_2_O content than conventional BG 45S5 (10.5 wt% vs. 24.5 wt%), additionally containing CaF_2_ (12 wt%) and a network connectivity similar to that of BG 45S5. A series of experimental composites functionalized with 5–40 wt% of the novel BG was prepared and compared to a corresponding series of experimental composites functionalized with 5–40 wt% of BG 45S5. Commercial acid-neutralizing materials (alkasite, giomer, and glass ionomer) were used as references. The capabilities of the materials to neutralize hydrochloric acid (pH = 2.6) and lactic acid (pH = 4.5) were evaluated by real-time pH measurements over 1 h. The degree of conversion and precipitation of calcium phosphate were also investigated. Data were analyzed using one-way and Welch ANOVA at an overall level of significance of 0.05. The acid-neutralizing potential of the experimental BG incorporated into resin composites was generally comparable to that of BG 45S5, and better than that of a giomer and glass ionomer. Fluorine was identified in the precipitate that developed on the composites functionalized with the experimental BG, suggesting a capability of forming fluorapatite. Unlike the 45S5 composition, the experimental BG did not impair the degree of conversion of resin composites. The novel BG filler is therefore an interesting candidate for future investigations of caries-preventive resin composites, and their potential clinical applicability for restorative, preventive, and orthodontic purposes.

## 1. Introduction

The fact that secondary caries is one of the primary reasons for the failure of composite restorations [1] has motivated intensive research of caries-inhibiting resin composites functionalized with bioactive glasses (BGs) [2,3,4,5,6,7]. In aqueous solutions, these reactive glasses release various ions, increase the pH, and precipitate calcium phosphate on their surfaces [8]. The reactivity of BGs is governed by the level of disruption within their structures, which are in turn determined by relative amounts of elements forming the glass network [9]. Using various compositional modifications, BG properties can be adjusted to fit a particular purpose, e.g., by releasing therapeutic ions or dissolving at a desired rate [10]. The conventional BG 45S5 is highly soluble due to its high sodium content, and the release of therapeutic ions is limited to calcium and phosphate [11]. In order to create a BG better suited for functionalizing resin composites, two compositional modifications of the conventional BG 45S5 were proposed: (i) a decrease in sodium content, rendering BG more stable and helping to maintain the mechanical properties of the composite, and (ii) an introduction of calcium fluoride, enabling the release of fluoride ions to support remineralization of dental hard tissues [3,4,5].

Modified BG compositions with lower sodium contents and added fluoride were investigated as reactive fillers in experimental resin composites and were shown to release calcium, phosphate, and fluoride ions [3], precipitate hydroxyapatite [4,5], and neutralize acid [6]. The acid-neutralizing capabilities of restorative materials were investigated by simulating lactic acid attack produced by biofilm bacteria (pH = 4.5) and hydrochloric acid attack due to regurgitation of gastric juice (pH = 2.6) [12,13]. The former type of acid attack has the potential to cause secondary caries, whereas the latter can affect marginal integrity of restorations [14,15]. Therefore, the capability of restorative materials to counteract these acid attacks would be beneficial for prolonging the service life of restorations. Besides inhibiting the dissolution of dental hard tissues mediated by low pH, the acid-neutralizing action of restorative materials can affect oral biofilms; raising the pH to neutral levels promotes a shift from acidogenic bacteria toward less cariogenic commensal flora [16], while increasing the pH to alkaline levels exerts antibacterial effects [17].

The degree of conversion (DC) represents a fundamental parameter governing mechanical properties and biocompatibility of polymer-based materials [18,19,20,21]. Previous studies indicated that BG 45S5 can impair the DC of experimental resin composites [22,23,24]. Since the present study is the first to investigate a particular novel BG composition, the possibility of it exerting a negative effect on DC was evaluated.

The formation of a calcium phosphate layer is an indicator of a material’s in vitro bioactivity [25]. Resin composite restorations capable of forming a calcium phosphate layer can seal marginal gaps and prevent bacterial penetration [26,27]. The calcium phosphate layer precipitated by conventional BG 45S5 is carbonated hydroxyapatite [9], whereas fluoride-modified BGs can precipitate a more acid-resistant fluorapatite layer [5]. This aspect was evaluated by comparing the calcium phosphate formation of a novel BG and conventional BG 45S5.

The aim of this study was to compare the effect of functionalizing resin composites with either a novel, fluoride-containing BG or conventional BG 45S5 on the following properties: (1) acid-neutralizing capability, (2) DC, and (3) precipitation of calcium phosphate. The null hypothesis assumed no effect of BG addition on the investigated properties.

## 2. Materials and Methods

### 2.1. Experimental and Reference Materials

The resin matrix for the experimental composites was prepared by mixing bisphenol-A-glycidyldimethacrylate (Bis-GMA, Merck, Darmstadt, Germany) and triethylene glycol dimethacrylate (TEGDMA, Merck) in a weight ratio of 60:40. The resin mixture was rendered photocurable by the addition of 0.2 wt% of camphorquinone (Merck) and 0.8 wt% of ethyl-4-(dimethylamino) benzoate (Merck). All components were mixed using a magnetic stirrer for 48 h.

BG 45S5, inert barium glass, and silica were obtained from commercial vendors. The experimental BG was prepared on-demand by the company Schott (Mainz, Germany) via the melt–quench route. The preparation and grinding procedures for the experimental BG were similar as for BG 45S5 in order to obtain similar particle sizes of both BG types. The experimental BG featured a lower Na_2_O content than conventional BG 45S5 (10.5 wt% vs. 24.5 wt%), and additionally contained 12 wt% of CaF_2_. The theoretical network connectivity of the experimental BG (2.1) was similar to that of conventional BG 45S5 [9]. Reinforcing fillers (inert barium glass and silica) were silanized, whereas the BG fillers were used without surface silanization.

Experimental composites were prepared by admixing varying ratios of bioactive and reinforcing fillers (Table 1) into the resin matrix. The series of composites containing 5–40 wt% of conventional BG 45S5 was denoted as the C-series, while the composite series functionalized with the same wt% of the experimental fluoride-containing BG was denoted as the E-series (Table 2). The control composite contained only reinforcing fillers. The total filler load in all composites was 70 wt%. The ratios of BG and reinforcing fillers followed previous studies of experimental BG-functionalized composites [23,24,28,29].

The resin system and the fillers were mixed using a dual asymmetric centrifugal mixing system (SpeedMixer DAC 150.1 FVZ, Hauschild and Co. KG, Hamm, Germany) at 2000 rpm. Mixing was performed in five one-minute intervals separated by one-minute breaks. After mixing, the prepared composites were deaerated in a vacuum for 48 h.

Three commercial acid-neutralizing materials were used as references, namely, a reinforced glass ionomer restorative (ChemFil Rock, Dentsply Sirona, Konstanz, Germany; shade: A2, LOT: 1807000740), a giomer (Beautifil II, Shofu, Kyoto, Japan; shade: A2, LOT: 041923), and a resin-based “alkasite” material (Cention, Ivoclar Vivadent, Schaan, Liechtenstein; shade: universal, LOT: XL7102). The alkasite material contained two types of reactive filers; an ionomer glass based on a calcium barium alumino-fluoro-silicate, and a calcium fluoro-silicate glass [27].

### 2.2. Acid Neutralization

**Figure 1:**
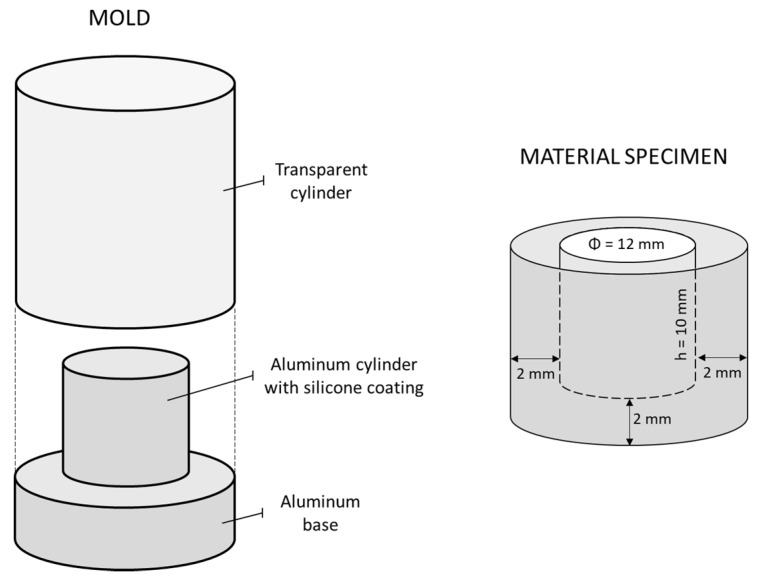
Schematic representation of the mold used to produce restorative material specimens (**left**); dimensions of the well within the material specimen (**right**).

The experimental protocol for real-time monitoring of the acid-neutralizing capabilities of restorative materials was modified according to a previous study [12], in which cylindrical wells were prepared within blocks of restorative materials using a CAD-CAM mill. To simplify specimen preparation and standardize surface roughness, the present study bypassed the use of a CAD-CAM mill by forming cylindrical wells directly within the specimens of unset materials using the mold shown in Figure 1. By impressing the base part of the mold into the transparent cylinder filled with the material, specimens with cylindrical wells were produced analogous to those prepared by milling in the original protocol [12]. The glass ionomer material was left to set in the mold for 15 min, while the light-curable materials (giomer and experimental composites) and the dual-curable alkasite material were illuminated using an LED curing unit (Bluephase PowerCure, Ivoclar Vivadent). This curing unit provided a radiant exitance of 1377 mW/cm^2^ in the wavelength range of 390–500 nm, as measured with a calibrated and NIST-referenced UV-vis spectrophotometer (MARC, BlueLight Analytics, Halifax, NS, Canada). The light guide tip of the curing unit was continuously moved around the transparent cylinder for 300 s to ensure complete polymerization of the large composite specimen. Due to the long curing time and overlapping illuminations, the outer specimen surface received a radiant exposure of at least 30 J/cm^2^ [30]. After removal from the mold, the inner surface of the well was additionally illuminated for 90 s (Bluephase PowerCure, Ivoclar Vivadent). The well surface was then ground for 3 min using P4000 silicon carbide (SiC) paper to standardize the surface roughness. Grinding was performed under dry conditions at a low speed (30 rpm) to avoid specimen heating. According to the particle size of the P4000 grinding paper (2.5 µm), the resulting surface roughness was comparable to that produced under clinical conditions by finishing the restoration with fine polishing disks [31]. After grinding, the composite specimens were copiously rinsed with deionized water for 2 min to remove the dust produced by grinding. The specimens were used immediately after preparation.

A hydrochloric acid solution (HAS) of pH = 2.6 and a lactic acid solution (LAS) of pH = 4.5 were prepared according to the previous study [12]. The wells within the composite specimens were filled with 600 µL of either HAS or LAS, and a magnetic stir bar was rotated at 300 rpm at the bottom of the wells. A calibrated pH electrode (780 pH Meter, Metrohm, Herisau, Switzerland) was immersed in the solution, and the environmental temperature was controlled at 23–24 °C. The pH values were logged with a resolution of 0.01 pH units every ten seconds for the first three minutes, then every minute for up to one hour. Five experimental runs were performed for each combination of the acid solution and restorative material (*n* = 5).

### 2.3. Degree of Conversion

For DC measurements of the experimental composites, discoid specimens (diameter = 6 mm, height = 2 mm) were prepared by casting uncured materials in polyoxymethylene (POM) molds, covering the mold openings with Mylar foil, and illuminating the composite cylinders from one side for 60 s (Bluephase PowerCure, Ivoclar Vivadent). After 15 min, the irradiated surface was dry-ground using P4000 SiC paper. This procedure mimicked the specimen preparation for acid neutralization measurements in order to produce a specimen surface of similar characteristics. The DC was evaluated 15 min after light curing by placing the ground specimen surface onto a diamond attenuated total reflectance (ATR) accessory of a Fourier transform infrared (FTIR) spectrometer (Cary 630 FTIR, Agilent Technologies, Santa Clara, CA, USA). The FTIR spectra were collected over 150 scans, using a resolution of 4 cm^−1^ in the wavelength range of 400–4000 cm^−1^. Eight experimental runs were performed for each material (*n* = 8). Spectra from uncured composites were recorded under the same conditions.

DC was calculated from the changes in the ratio of absorbance intensities (peak heights) of aliphatic C=C (1638 cm^−1^) and aromatic C⋯C (1608 cm^−1^) spectral bands using the following equation [32]:

(1) $$\mathrm{DC}\;\left(\%\right)=\left(1\;-\;\frac{{(1638\;\mathrm{cm}}^{-1}{/1608\;\mathrm{cm}}^{-1})\;\mathrm{after}\;\mathrm{curing}}{{(1638\;\mathrm{cm}}^{-1}{/1608\;\mathrm{cm}}^{-1})\;\mathrm{before}\;\mathrm{curing}}\right)\;\times \;100.$$

### 2.4. Scanning Electron Microscopy and Energy-Dispersive X-Ray Spectroscopy

Discoid specimens (d = 6 mm, h = 2 mm) were prepared from the control, C-40, and E-40 composites. Specimen preparation followed the procedure described above for the DC measurements. Composite specimens were immersed separately for 28 days in 5 mL of phosphate-buffered saline (PBS, pH: 7.4; Gibco, Life Technologies, Carlsbad, CA, USA) at 37 °C. After the immersion period, specimens were rinsed with distilled water, dried, and sputtered with carbon. A scanning electron microscope (SEM; GeminiSEM 450, Zeiss, Oberkochen, Germany) was used at 3 kV and 20,000× magnification to evaluate the precipitate formed on the composite surfaces after immersion in PBS. The energy-dispersive X-ray spectroscopy (EDX) analysis was performed at 10 kV using the X-ray detector X-MAX80 (Oxford Instruments, Abingdon, UK).

### 2.5. Statistical Analysis

Normality of distribution was verified using Shapiro–Wilk’s test. Due to a significant nonuniformity of variances indicated by Levene’s test for the pH values measured after 1 h and the time required to reach a pH of 5.5, intermaterial comparisons for these variables were performed using a robust (Welch) ANOVA with Games–Howell post-hoc adjustment. Mean values for the variables with uniform variances (extent of pH increase and DC) were compared among materials using a one-way ANOVA with Tukey post-hoc adjustment. The statistical analysis was performed using SPSS (version 20, IBM, Armonk, NY, USA) at an overall level of significance of α = 0.05.

## 3. Results

**Figure 2:**
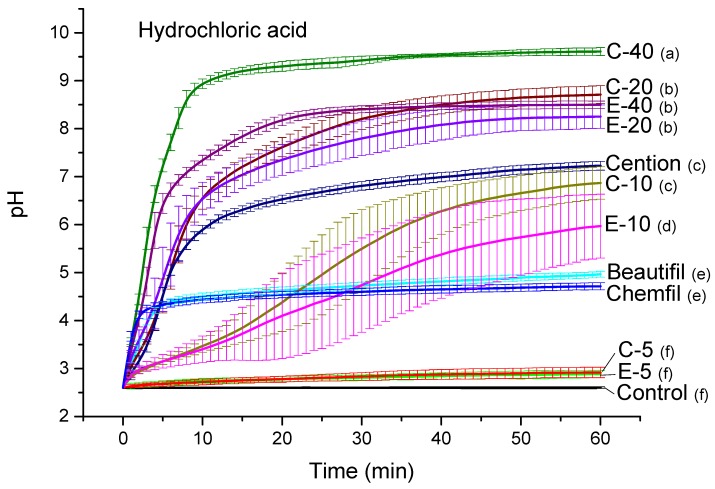
pH of hydrochloric acid solution (mean values ± standard deviation). Different letters denote significantly different pH values at the end of the measuring period (*p* < 0.05).

Curves of the pH in HAS are shown in Figure 2. The final pH values reached by experimental composites ranged between 2.9 and 9.6, indicating a significant improvement in acid-neutralizing capability with increasing BG amounts (*p* < 0.001). Experimental materials with 5 wt% of BG showed negligible acid neutralization, similar to that of the control composite (*p* = 0.65–0.78). The glass ionomer and the giomer showed similar acid-neutralizing potentials, reaching final pH values of 4.7 and 5.0, respectively (*p* = 0.88). Experimental composites with 10–40 wt% of BG showed better acid-neutralizing capabilities than the glass ionomer and giomer (*p* < 0.001). The neutralizing capability of the alkasite material was similar to that of the experimental composite functionalized with 10 wt% of BG 45S5 (*p* = 0.45).

**Figure 3:**
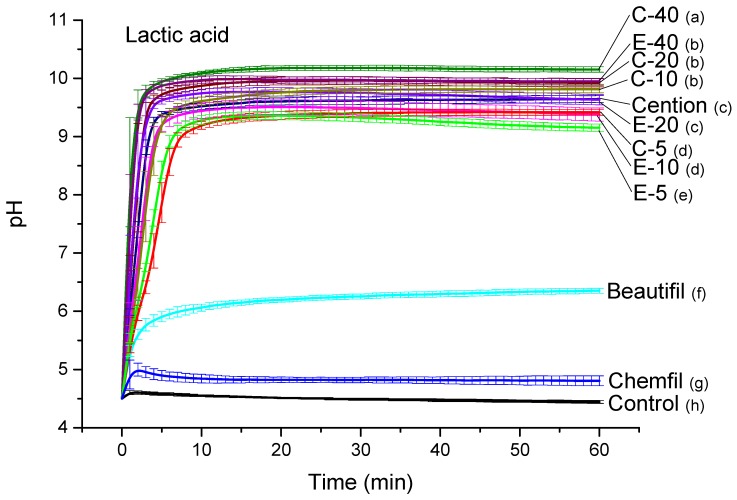
pH of lactic acid solution (mean values ± standard deviation). Different letters denote significantly different pH values at the end of the measuring period (*p* < 0.05).

Curves of the pH rise in LAS are shown in Figure 3. The acid-neutralizing capabilities of experimental composites improved with higher BG amounts (*p* < 0.001). However, the influence of the BG amount on acid neutralization was less pronounced than in HAS, as evidenced by a narrower range of final pH values for experimental composites (9.2–10.1). This pH range was comparable to the pH reached by the alkasite (9.6, *p* = 0.99) and significantly higher than the pH values reached by the giomer (6.4, *p* < 0.001) and glass ionomer (4.8, *p* < 0.001).

**Figure 4:**
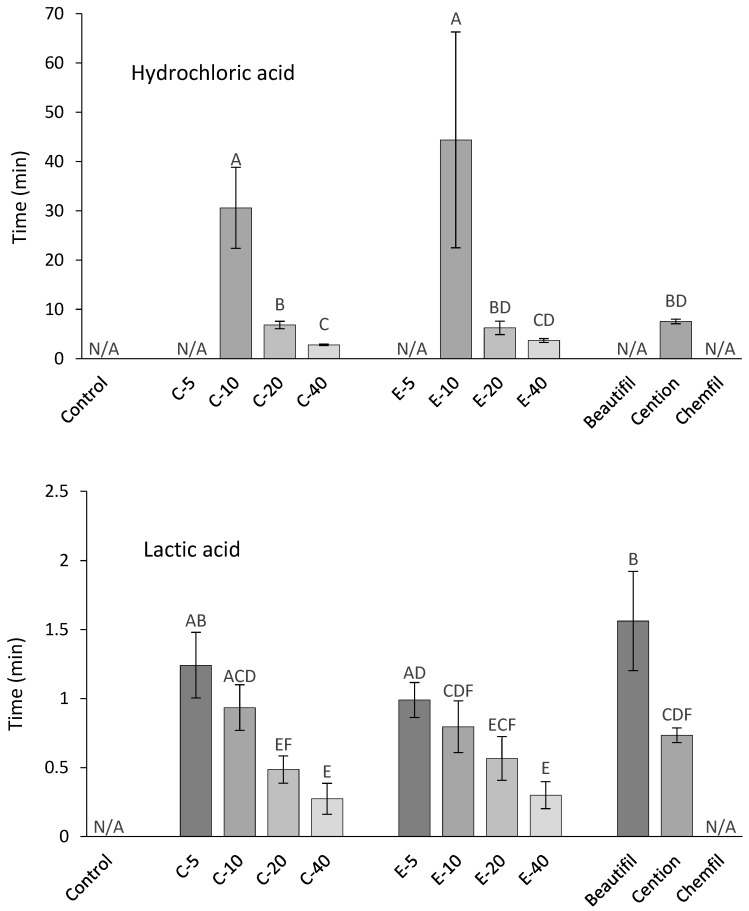
Time required for hydrochloric and lactic acid solutions to reach a pH of 5.5 (mean values ± standard deviation). Cases of a material failing to reach a pH of 5.5 are denoted as the parameter being not available (N/A). Different letters denote significantly different values within an acid solution (*p* < 0.05).

As a measure of acid-neutralization rate, the time required for the solutions to reach pH = 5.5 is shown in Figure 4. In both HAS and LAS, the rate of acid neutralization was improved by higher BG amounts (*p* < 0.001) but remained unaffected by the type of BG (*p* = 0.16–0.17). The alkasite showed a similar acid-neutralization rate as experimental composites with 20 wt% of BG (*p* = 0.49–0.89). In LAS, the giomer neutralized acid slower than the experimental composites with 10–40 wt% of BG (*p* < 0.001). A pH of 5.5 was not reached by the giomer in HAS nor by the glass ionomer in either acid solution.

**Figure 5:**
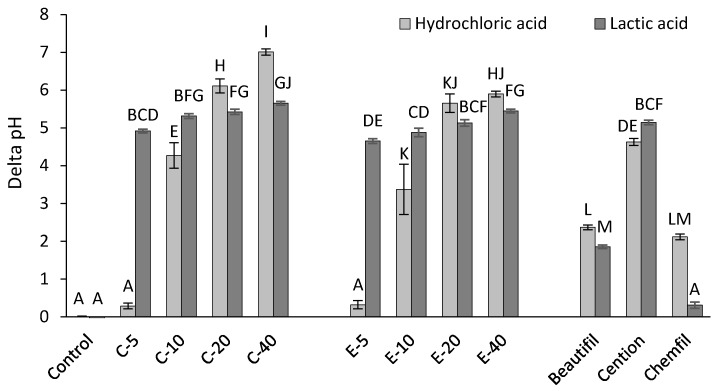
pH increase (Δ pH) in hydrochloric and lactic acid solutions (mean values ± standard deviation). Different letters denote significantly different values (*p* < 0.05).

The extent of pH increase during the measuring period of 1 h is shown in Figure 5. The pH increases of experimental composites containing 10 wt% or 20 wt% of BG were similar to that of the alkasite in LAS (*p* = 0.69–0.99), and higher than that of the glass ionomer and the giomer, irrespective of the acid solution (*p* < 0.001). The pH increases of experimental composites in LAS existed in a narrower range (4.7–5.7 pH units) compared to HAS (0.3–7.0 pH units). Composites functionalized with experimental BG showed similar or lesser pH increases compared to the corresponding composites functionalized with BG 45S5.

**Figure 6:**
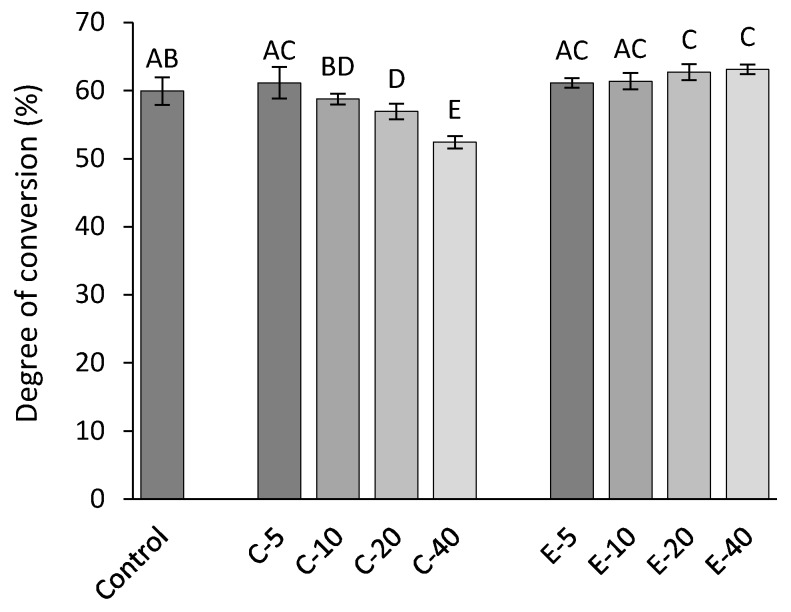
Degree of conversion of experimental composites (mean values ± standard deviation). Different letters denote significantly different values (*p* < 0.05).

Figure 6 shows the DC of experimental composites. The composite series functionalized with conventional BG 45S5 showed a statistically significant DC decline with increasing BG amounts (*p* < 0.001), whereas the composite series functionalized with experimental BG showed similar or improved DC values in comparison to the control composite.

**Figure 7:**
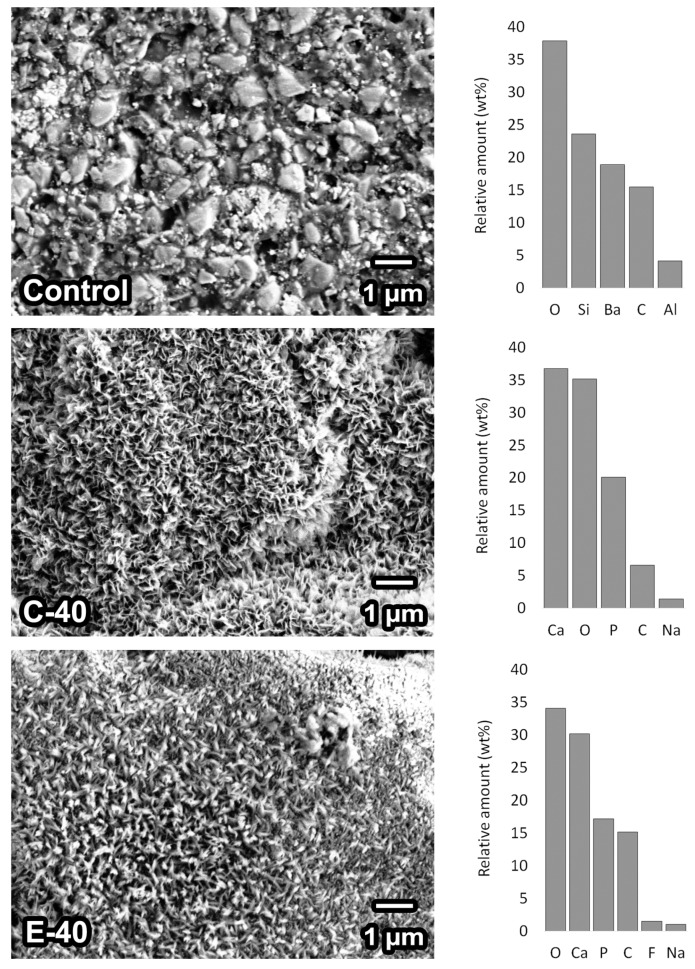
Scanning electron microscopy images of composite surfaces after 28 days of storage at 37 °C in phosphate-buffered saline (PBS). The energy-dispersive X-ray spectroscopy data are represented by relative amounts (in wt%) of elements identified on specimen surfaces after PBS immersion.

Figure 7 shows SEM images and the results of the EDX analysis for composite surfaces after 28 days in PBS. Glass particles of approximately 1 µm and comparatively smaller agglomerates of silica were visible on the surface of the control composite. The composites C-40 and E-40 were covered by a crystalline precipitate with Ca/P molar ratios of 1.41 and 1.36, respectively. For the composite E-40, the presence of fluorine in the precipitate was identified by EDX analysis.

## 4. Discussion

This study showed that an experimental low-sodium, fluoride-containing BG admixed into a Bis-GMA/TEGDMA resin matrix presented an acid-neutralizing potential generally comparable to that of conventional BG 45S5. Experimental composites functionalized with either BG type showed acid-neutralizing capabilities superior to those of the giomer and the glass ionomer, as well as the nonfunctionalized composite. The DC was diminished by the addition of conventional BG 45S5, but not by the experimental BG. Upon immersion in PBS, the composites containing either type of BG formed calcium phosphate layers, with fluorine incorporated into the precipitates of the composites functionalized with the experimental BG. These results led us to reject the null hypothesis.

The experimental BG in this study was prepared by modifying the conventional BG 45S5 composition by introducing 12 wt% of CaF_2_. Adding CaF_2_ by partly replacing Na_2_O or CaO was shown to increase the network connectivity of BG and consequentially diminish its reactivity [33]. However, in this study, reducing the amount of Na_2_O from 24.5 wt% to 10.5 wt% was accompanied by adjusting the relative ratios of other elements (Table 1) to maintain network connectivity at the level of BG 45S5. These compositional modifications helped to maintain optimal reactivity of the experimental BG [34].

As caries is basically caused by the dissolution of dental hard tissues under low pH conditions, the acid-neutralizing capabilities of various commercial and experimental restorative materials was investigated as a potential means for preventing secondary caries [3,12,35,36,37,38,39]. Due to the lack of a standardized procedure for evaluating acid-neutralizing potential, these studies were performed under a wide range of different conditions. As a means of standardizing the experimental protocol, some studies maintained a constant ratio of tested material volume to immersion solution volume [37,40,41]. However, for short-term measurements (up to several hours), the volume of solution per material surface area may be more meaningful, as the bulk of a specimen cannot be permeated by a solution within such a short time frame [42] and the neutralization reaction mostly occurs on a specimen’s surface.

Similar to other laboratory studies on acid-neutralizing capabilities [3,37,40,41], the solution volume used in the present study largely overestimated the amount of acid required to be neutralized under clinical conditions. Other features of clinical situations that cannot be reliably simulated in laboratory studies involve the inflow of fresh saliva, which possesses its own buffering properties [43], as well as the intermittent production of new acid by microbial flora. Acknowledging these unavoidable limitations of in vitro studies, it should be noted that using higher amounts of acid solution combined with no inflow of fresh saliva overexaggerates the acidic attack and creates more demanding conditions than those occurring in marginal gaps [39]. This consideration was conveniently demonstrated in a study that compared the acid neutralization capabilities of restorative materials within bulk solutions and thin films, representing laboratory and clinical conditions, respectively [38]. Therefore, although the data from laboratory studies cannot be extrapolated to clinical situations in absolute terms, it is plausible that acid neutralization would be more extensive and faster in the latter case.

Measurements using HAS showed a higher discriminative potential for acid-neutralizing capabilities, with final pH values of experimental composites being in the range of 2.9–9.6 compared to the corresponding pH values of LAS in the range of 9.2–10.1. This difference in discriminative potential was noted for both the BG amount (higher BG amounts neutralized better) and BG type (BG 45S5 tended to neutralize better than experimental BG). However, the rankings of experimental composites were similar in both solution types. In an experimental setup similar to ours, HAS of pH = 2.6 also discriminated better between restorative material types (composites, compomer, glass ionomer, and giomer) than LAS of pH = 4.5 [12]. Although both types of acid solution represent a clinically realistic acid attack, LAS of pH = 4.6 seems more suitable for evaluating the caries-inhibiting potential of experimental materials, whereas HAS of pH = 2.5 could be used to stress materials under extremely acidic conditions in order to better discriminate between different material compositions.

The alkasite and the experimental composites (except those with 5 wt% of BG in HAS) neutralized acid better than the glass ionomer and the giomer, indicating a higher acid-neutralizing potential of various types of alkaline bioactive glasses compared to reactive fillers found in the glass ionomer and the giomer. Although the high initial reactivity implied faster exhaustion of water-soluble fillers, some evidence exists that composites functionalized with low-sodium, fluoride-containing BG can maintain their alkalizing potential for up to 180 days [3]. Future work will evaluate the sustainability of this acid-neutralizing potential, as well as its protective effect on dental hard tissues under conditions of intermittent acid exposure.

Under physiological conditions, it takes about 30 min for saliva to neutralize lactic acid produced by biofilm bacteria [44]. By neutralizing lactic acid faster than saliva, bioactive restorative materials can shorten the time available for demineralization, thereby exerting a protective effect on dental hard tissues. The fact that all experimental composites reached alkaline pH values within several minutes in LAS indicated that a clinically useful neutralization of a moderate acidic solution (pH = 4.5) may be attained by functionalizing resin composites with BG amounts as low as 5 wt%. Limiting the amount of reactive fillers to the lowest level sufficient for bioactivity could help to maintain the mechanical properties of the composite [28]. However, if the composites are intended to neutralize a more aggressive acid attack, such as that of HAS with a pH of 2.6, the more pronounced influence of BG amount on neutralizing capability should be considered. It should also be noted that the acid-neutralizing capabilities evaluated in the present study corresponded to freshly prepared specimens and that slower and less extensive acid neutralization should be expected in aged materials.

In order to compare acid neutralization rates among different materials, the time required to reach a pH of 5.5 was calculated [37]. This parameter was used as a relative measure of acid neutralization rate and was not intended to simulate the time needed to arrest the dissolution of dental hard tissues under clinical conditions. However, according to the previously discussed differences between laboratory and clinical conditions, this parameter was likely to overestimate the time frame required for acid neutralization within marginal gaps. Therefore, the rapid pH increase induced by all experimental composites in LAS (up to pH = 5.5 in less than 1.5 min) implies that even faster acid neutralization may occur clinically. The neutralization rates of experimental composites in LAS were generally similar or higher than those of the giomer and alkasite materials, whereas the glass ionomer failed to reach a pH of 5.5. In HAS, the intermaterial differences in neutralization rates were more pronounced, with times required to reach pH = 5.5 ranging between 2.8 and 44.4 min. This difference was expected, as a concentration of protons almost 80 times greater than in LAS was required to be neutralized in HAS to reach a pH of 5.5. This also explains why the giomer, glass ionomer, and experimental composites with 5 wt% of BG failed to reach pH = 5.5 when exposed to this highly acidic solution.

Since BG reactivity increases at low pH values, greater pH increase was expected in HAS (pH = 2.6) than in LAS (pH = 4.5) [3,12]. However, inverse results were observed for the experimental composites with 5 wt% and 10 wt% of BG, as well as for the commercial alkasite material. A similar finding was reported for a resin composite functionalized with nanofluorapatite [12]. The lower BG loadings incorporated here provided only a small amount of exchangeable Na^+^ ions, thus, the potential to raise the pH was limited compared to the high loadings of up to 40 wt%. As hydrochloric acid is a strong and fully dissociated acid, the pH rise by the less alkalized material (low BG loading) was very limited compared to that expected of a weak acid, such as LAS, at which the delta pH can be higher.

In a previous study, a composite functionalized with 10 wt% of nano-sized bismuth-modified BG 45S5 reached a pH value of 9.2 in HAS [12]. We obtained alkaline pH values (8.2–9.6) in HAS only for the composites functionalized with 20 wt% and 40 wt% of BG, while the experimental composites with lower BG amounts reached significantly lower pH values (2.9–6.9). This difference in alkalizing capability in HAS can be attributed to the higher reactive surface area of nano-sized BG compared to the micro-sized BG used in the present study. However, the pH of 9.8 measured for the composite containing nano-sized BG in LAS [12] was similar to the final pH values in LAS reached by all of our BG-containing composites (9.2–10.1). This observation implies that the effect of BG particle size becomes more pronounced in solutions of higher acidity. Although highly reactive nano-sized BG fillers appear to be beneficial for neutralizing more aggressive acidic attacks, the limitations related to the effect of the high surface area of nanoparticles on material viscosity should be considered when designing novel bioactive composites [45].

As Na^+^ are the main ions responsible for an exchange process with protons (H^+^) from the solution, the Na_2_O content determines the alkalizing capability of a particular BG composition [46]. A fluoride-modified BG composition containing less Na_2_O than that in the present study (6 mol% vs. 11 mol%) was recently investigated as a reactive filler for resin composites [3]. Composites in the aforementioned study showed acid-neutralizing potential by increasing the pH from 4 to 7; however, the pH increase in a neutral solution was less extensive (from 7 to around 8.5). Given the long immersion time in that study (180 days), it appears that a pH of 8.5 was the maximum value attainable by the particular BG composition. Compared to the more alkalizing experimental BG investigated in this study (maximum pH values of 9.2–10.1), BG compositions with lower alkalizing potentials may feature slower degradation in an acidic environment and longer-lasting protection of dental hard tissues [5]. Furthermore, restricting the BG reactivity to the acidic range without extending it into the alkaline region may help in the design of responsive materials, which would react in a low-pH environment but conserve their reactive potential in neutral-pH solutions. On the other hand, a more reactive BG formulation offers the benefit of faster acid neutralization and the antibacterial effects of the alkaline environment [17]. It is as yet unclear which of these two behaviors would be more beneficial for secondary caries prevention, limited alkalization maintained over a longer period of time, or stronger alkalization at the cost of earlier depletion.

The glass ionomer showed surprisingly poor acid-neutralizing capabilities in LAS, with results comparable to those of the control composite. Similar behavior was reported previously for another commercial glass ionomer material, which neutralized LAS at a pH of 4.5 to a lesser extent than all other materials, including conventional composites with no reactive fillers [12]. The glass ionomer in that study neutralized significantly better in HAS (pH = 2.6), reaching a pH value comparable to that of the giomer. This observation is consistent with the results of our study, in which the glass ionomer neutralized better at lower pH levels and performed similarly to the giomer. Since the setting reaction of the glass ionomer was still at an early stage at the time of the pH measurements (15 min after mixing), it is possible that the high acidity of the freshly mixed material limited its acid-neutralizing potential to pH = 4.8. The pH curves plateauing at the same pH value of 4.8 in both HAS and LAS supported the explanation that this was the maximum pH attainable by the freshly mixed materials. Considering the data from other studies, conflicting results exist for the acid-neutralizing potential of glass ionomer materials. For example, for resin-modified glass ionomers immersed in LAS (pH = 4.0), one study showed the potential to raise the pH up to 6.2 [35], while another study [37] reported virtually no induction of pH changes in LAS (pH = 4.0). In addition, a study investigating the pH rise of four glass ionomers in LAS (pH = 2.6) reported their potential to increase the pH to 4.1–4.6 [39]. There is also a report of a low acid-neutralizing potential of a glass ionomer that was not improved by aging the specimens for 24 hours [12]. From such inconsistent results, the acid-neutralizing potential of glass ionomers appears to be highly dependent on material composition and experimental conditions.

Previous studies showed that the DC of resin composites could be impaired by the addition of BG 45S5 [22,23,24]. This phenomenon was confirmed in the present study for the composite series functionalized with BG 45S5, which showed a dose-dependent DC decline. In contrast, no negative effects on DC were identified for composites containing the experimental BG. The composites with 20 wt% and 40 wt% of the experimental BG even showed a significantly improved DC compared to that of the control composite, which can be attributed to improved resin mobility due to the replacement of nano-sized silica with micro-sized BG fillers [47]. The sensitivity of the polymerization reaction to the inhibitory potential of BG 45S5 was previously shown to depend on the composition of the resin system [22], whereas the results of the present study indicated that the BG composition itself can affect its inhibitory potential. Although DC differences can theoretically affect the acid-neutralizing capability of composites by determining water diffusion through the polymeric network [48], it is unknown on which time-scale this effect becomes detectable. However, the results of the present study point to a more relevant fact, namely that up to 40 wt% of experimental BG could be added to a Bis-GMA/TEGDMA resin matrix without diminishing its DC. Maintaining high DC values is welcome in order to avoid unnecessarily impairing the mechanical properties, which tend to be jeopardized by the addition of soluble fillers [28].

The potential of the experimental BG for precipitating calcium phosphate was compared to that of BG 45S5 by performing SEM and EDX examinations on composites containing 40 wt% of BG fillers after 28 days of PBS immersion. Both types of BG formed precipitates of similar morphologies, and molar Ca/P ratios characteristic for calcium-deficient hydroxyapatite. Although an in-depth analysis of the precipitate was outside the scope of the present study, the presence of fluorine in the precipitate formed by the composite E-40 implies the formation of fluorapatite. As fluorapatite is less soluble than hydroxyapatite, the precipitate formed by the experimental fluoride-modified BG may provide a more durable marginal gap sealing compared to the precipitate formed by conventional BG 45S5 [5]. Moreover, the remineralization of dental hard tissues may be improved by the availability of fluoride ions [49]. The ions released by BG-functionalized composites can act synergistically with the acid-neutralizing effect to protect dental hard tissues from mineral loss. As the concentrations of calcium, phosphate, and fluoride ions in the solution increase, the equilibrium shifts toward remineralization, even under acidic pH conditions [50]. The combined benefits of these effects for protecting dental hard tissues from demineralization will be addressed in a future study.

Despite the limitations discussed above (overestimating the amount of acid to be neutralized, lack of intermittent acid production, and no inflow of fresh medium with buffering properties), this study demonstrated the potential of BG-functionalized resin composites for protecting dental hard tissues, which could be useful for different clinical dental applications. In restorative dentistry, protection against secondary caries can prolong the service life of composite restorations [7]. BG-functionalized fissure sealants can be used in preventive dentistry [6], whereas orthodontic adhesives containing BG fillers can help to prevent the occurrence of white spot lesions [3,4,5].

## 5. Conclusions

The acid-neutralizing potential of the experimental low-sodium, fluoride-containing bioactive glass incorporated into resin composites was generally comparable to that of the conventional 45S5 composition, and better than that of a giomer and glass ionomer. Fluorine identified in the calcium phosphate precipitated by composites functionalized with the experimental bioactive glass suggests that they possess capabilities to form fluorapatite. Unlike the 45S5 composition, the experimental bioactive glass did not impair the degree of conversion of resin composites. These beneficial features make the novel bioactive glass an interesting candidate for future investigations of caries-preventive resin composites and indicate potential clinical applicability for restorative, preventive, and orthodontic purposes.

## Figures and Tables

**Table 1:** Compositional details of bioactive glass and reinforcing fillers used in experimental composites.

	Bioactive Glass 45S5	Experimental Fluoride-Containing Bioactive Glass	Inert Barium Glass	Silica
Particle size (d50)	3 µm	3 µm	1 µm	5–50 nm
Composition (wt%)	45.0% SiO_2_ 24.5% CaO 24.5% Na_2_O 6.0% P_2_O_5_	33.5% SiO_2_ 33.0% CaO 10.5% Na_2_O 11.0% P_2_O_5_ 12.0% CaF_2_	55.0% SiO_2_ 25.0% BaO 10.0% Al_2_O_3_ 10.0% B_2_O_3_	>99.8% SiO_2_
Silanization (wt%)	none	none	3.2	4–6
Manufacturer	Schott, Mainz, Germany	Schott, Mainz, Germany	Schott, Mainz, Germany	Evonik, Hanau, Germany
Product name/LOT	G018-144/M111473	experimental batch	GM27884/Sil13696	Aerosil R 7200/157020635

**Table 2:** Composition of experimental composites.

Material Designation		Filler Composition (wt%)			Total Filler Ratio (wt%)
		Bioactive Glass 45S5	Experimental Fluoride-Containing Bioactive Glass	Reinforcing Fillers (Inert Barium Glass: Silica = 2:1)	
	**Control**	0	0	70	70
C-series	**C-5**	5	0	65	70
	**C-10**	10	0	60	70
	**C-20**	20	0	50	70
	**C-40**	40	0	30	70
E- series	**E-5**	0	5	65	70
	**E-10**	0	10	60	70
	**E-20**	0	20	50	70
	**E-40**	0	40	30	70
